# Familiarity Affects Entrainment of EEG in Music Listening

**DOI:** 10.3389/fnhum.2017.00384

**Published:** 2017-07-26

**Authors:** Yuiko Kumagai, Mahnaz Arvaneh, Toshihisa Tanaka

**Affiliations:** ^1^Department of Electrical and Electronic Engineering, Tokyo University of Agriculture and Technology Koganei-shi, Japan; ^2^Department of Automatic Control and Systems Engineering, University of Sheffield Sheffield, United Kingdom; ^3^RIKEN Brain Science Institute Wako-shi, Japan

**Keywords:** music, entrainment, perception, electroencelphalogram (EEG), spectrum analysis

## Abstract

Music perception involves complex brain functions. The relationship between music and brain such as cortical entrainment to periodic tune, periodic beat, and music have been well investigated. It has also been reported that the cerebral cortex responded more strongly to the periodic rhythm of unfamiliar music than to that of familiar music. However, previous works mainly used simple and artificial auditory stimuli like pure tone or beep. It is still unclear how the brain response is influenced by the familiarity of music. To address this issue, we analyzed electroencelphalogram (EEG) to investigate the relationship between cortical response and familiarity of music using melodies produced by piano sounds as simple natural stimuli. The cross-correlation function averaged across trials, channels, and participants showed two pronounced peaks at time lags around 70 and 140 ms. At the two peaks the magnitude of the cross-correlation values were significantly larger when listening to unfamiliar and scrambled music compared to those when listening to familiar music. Our findings suggest that the response to unfamiliar music is stronger than that to familiar music. One potential application of our findings would be the discrimination of listeners' familiarity with music, which provides an important tool for assessment of brain activity.

## 1. Introduction

When listening to music, a human perceives beats, meters, rhythms, melodies, and so on. It has been reported that music perception involves emotion, syntactic processing, and motor system (Maess et al., 2001; Pereira et al., 2011; Koelsch et al., 2013). For example, Koelsch et al. (2013) observed brain connectivity between visual cortex and area seven of the superior parietal lobule when participants perceived auditory signals of danger. Maess et al. (2001) showed that brain areas involved in language syntactic analysis was activated during musical syntactic processing. Interestingly, Pereira et al. (2011) showed that passive listening to music in non-musicians led to motor cortex activation. Despite all these studies, the mechanism of music perception is still unclear.

To understand auditory mechanism many researchers measure event-related potentials (ERPs) such as mismatch negativity (MMN) in numerous contexts in the music domain and in the speech domain. MMN is a change-specific component of ERP that has a peak at 150–250 ms after the onset of deviant stimulus (Näätänen et al., 1978). Some research studies have shown that MMNs are elicited by the deviant sound in rhythmic sequences (Lappe et al., 2013), melody (Virtala et al., 2014), and speech (Dehaene-Lambertz, 1997). Another approach in understanding auditory mechanism is to investigate auditory steady-state response (ASSR) which can be elicited by periodically repeated sounds (Lins and Picton, 1995). It has been reported that in speech perception domain amplitude-modulated speech could elicit ASSR (Lamminmäki et al., 2014). Interestingly, recent investigations in music perception domain have demonstrated that ASSR was evoked by periodic rhythm of music (Meltzer et al., 2015). However, the MMN and ASSR approaches are not suitable for stationary stimuli such as natural music.

Recent works on speech perception have focused on phase entrainment (Ahissar et al., 2001; Luo and Poeppel, 2007; Aiken and Picton, 2008; Nourski et al., 2009; Ding and Simon, 2013, 2014; Doelling et al., 2014; Zoefel and VanRullen, 2015, 2016). Cortical entrainment to the envelope of speech has been investigated by using magnetoencephalogram (MEG) (Ahissar et al., 2001), electroencelphalogram (EEG) (Aiken and Picton, 2008), and electrocorticogram (ECoG) (Nourski et al., 2009). Many researchers reported that cortical entrainment was correlated with the speech intelligibility (Ahissar et al., 2001; Luo and Poeppel, 2007; Aiken and Picton, 2008; Ding and Simon, 2013; Doelling et al., 2014). Moreover, it has been suggested that intelligible speech could enhance the entrainment compared to unintelligible speech (Luo and Poeppel, 2007; Doelling et al., 2014; Zoefel and VanRullen, 2015). Thus, high-level factors of speech sound which reflect intelligibility could play an important role in cortical entrainment.

In the music perception domain, cortical entrainment to periodic stimuli such as beat, meter, and rhythm has been observed in many studies (Fujioka et al., 2012; Nozaradan, 2014; Meltzer et al., 2015). Recently, it was demonstrated that cerebral cortex entrains to the music by using MEG (Doelling and Poeppel, 2015). Moreover, some researchers have investigated the relationship between entrainment and emotion while listening to music in different contexts (Trost et al., 2017). For instance, using functional magnetic resonance imaging (fMRI) it has been shown that emotion and rhythm of music affect the entrainment (Trost et al., 2014). Since music includes complex features such as rhythm, melody, and harmony, the link between entrainment and high-level factors is still open to question.

Music familiarity is an important high-level factor in music perception. There are many brain imaging studies focusing on brain regions activated by familiar music, such as (Satoh et al., 2006; Groussard et al., 2009; Pereira et al., 2011), however, they did not investigate entrainment. In EEG studies, it was shown that a deviant tone among a sequence of familiar tones enhanced MMN compared to that among a sequence of unfamiliar sounds (Jacobsen et al., 2005), and deviant chord among a sequence of familiar chord elicited a greater response than that among a sequence of unfamiliar chord (Brattico et al., 2001). Another study reported that the cerebral cortex responded more strongly to the periodic rhythm of unfamiliar music than to that of familiar music (Meltzer et al., 2015). Regardless of these interesting findings, as mentioned above, it has not been clarified how the familiarity of music affects the response of the cortical entrainment.

In this study, we investigated the difference of cortical response depending on familiarity of music focusing on cortical entrainment. Since recent speech perception studies demonstrated high-level factors affecting entrainment, we hypothesized that entrainment to music would be influenced by familiarity which is one of the high-level factors of music perception. To test this hypothesis, we calculated cross-correlation function between the envelope of the played music and EEG recorded during listening to three kinds of music i.e., familiar, unfamiliar, and scrambled.

## 2. Materials and methods

### 2.1. Participants

Eight males (mean age 22.4 ± 0.744, range 21 – 23 year old) who had no professional music education participated in this experiment. All participants were healthy; none reported any history of hearing impairment or neurological disorder. They were signed an informed consent form. The study was approved by the Human Research Ethics Committee of the Tokyo University of Agriculture and Technology.

### 2.2. Task and stimuli

#### 2.2.1. Sound stimuli

**Figure 1:**
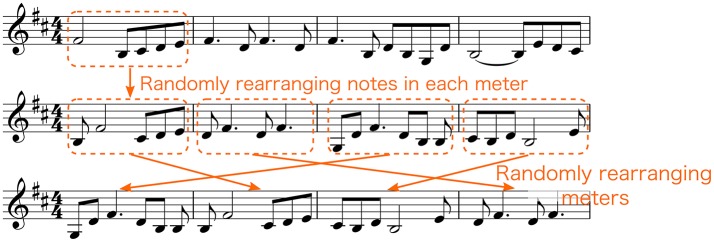
Procedure of creating scrambled versions of sound stimuli. Notes in each meter were randomized. Thereafter, the meters were randomized.

We extracted two types of sound stimuli, original and scrambled versions, using the music computation and notation software called Sibelius (Avid Technology, USA). We created 20 pieces of the original version that consisted of melodies produced by piano sounds as shown in the Table 1. We then created 10 pieces of the scrambled version using the upper 10 pieces of Table 1 by randomizing notes in each meter, and then randomizing the order of the meters (Figure 1). Randomization was implemented through a custom-written Python program that operated on an XML file generated in Sibelius. Thus, in total, we prepared 30 musical pieces. The length of each musical piece was 32 s with the tempo set to 150 beat per minute (bpm) (i.e., frequency of a quarter of a note was 2.5 Hz). The sampling frequency was set to 44,100 Hz, and resampled to 32,768 Hz for analysis. Examples of sound stimuli can be found in Supplementary Materials.

**Table 1:** Music for sound stimuli.

**Composer**	**Title**	**Familiar/unfamiliar**
Popular English lullaby	Twinkle Twinkle Little Star	8/0
A. L. Vivaldi	The Four Seasons, Spring	8/0
P. I. Tchaikovsky	The Nutcracker, March	7/1
P. I. Tchaikovsky	Swan Lake, Scene	8/0
A. Dvorak	Symphony No. 9 “From The New World”	8/0
T. Hakase	Jounetsu Tairiku	8/0
A. Khachaturyan	Masquerade	3/5
J. Pachelbel	Canon	7/1
L. v. Beethoven	Ode to Joy	8/0
W. A. Mozart	Eine Kleine Nachtmusik	8/0
I. Albeniz	Piano Sonate Op.82	0/8
F. Kuhla	Sonatine Op.55-1	0/8
A. Diabelli	Sonatine Op.151-2	0/8
A. Diabelli	Sonatine Op.168-2	2/6
P. I. Tchaikovsky	Six Pieces Op.51-1	1/7
G. Faure	Dolly Suite, Kitty-valse	0/8
L. v. Beethoven	Piano Sonate Op.14-1	0/8
L. v. Beethoven	The Creatures of Prometheus, Introduction	0/8
F. Mendelssohn	Lieder Ohne Worte Op.19-1	0/8
W. A. Mozart	Piano Sonate KV309	0/8

*Twenty pieces of the original version were extracted based on the music mentioned in this table. Ten pieces of the scrambled version were created based on the upper 10 pieces of this table. Third column shows the number of participants that rated each piece as familiar and unfamiliar*.

#### 2.2.2. Task procedure

**Figure 2:**
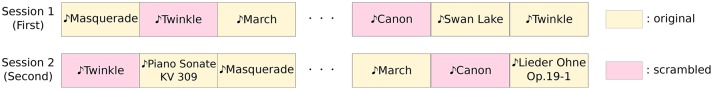
An experimental paradigm. The experiment consisted of two sessions, and each session was divided into thirty trials. In each trial EEG recordings, 34 s in duration, were acquired. Each of the thirty trials employed a different sound stimuli at random.

In the whole experiment, participants listened to the sound stimuli while visually fixating at a stationary position. An experimental paradigm is shown in Figure 2. The experiment consisted of two sessions where each session included 30 trials. In each trial, EEG recordings, 34 s in duration, were acquired while the participant was listening to one of the 30 created melodies. At the end of each trial, the participants were asked whether they were familiar with the presented melody. It is noted that the order of the sound stimuli was random in each session. After each session, the EEG recordings of all the trials were assessed for detecting artifacts such as large-amplitude spikes. Each trial was visually inspected during the experiment. If the trial was contaminated with a large amount of artifacts, it was not added to the recording dataset and the corresponding trial was repeated to the participants.

After the experiment, for each participant, the original version of stimuli were categorized into two groups (familiar and unfamiliar) according to the answer of the participant. Trials which participant's answers were familiar were labeled as familiar, and trials which participant's answers were unfamiliar were labeled as unfamiliar. If the participant's answers were not consistent across the sessions, the corresponding trials were excluded from the recordings.

### 2.3. EEG data acquisition

**Figure 3:**
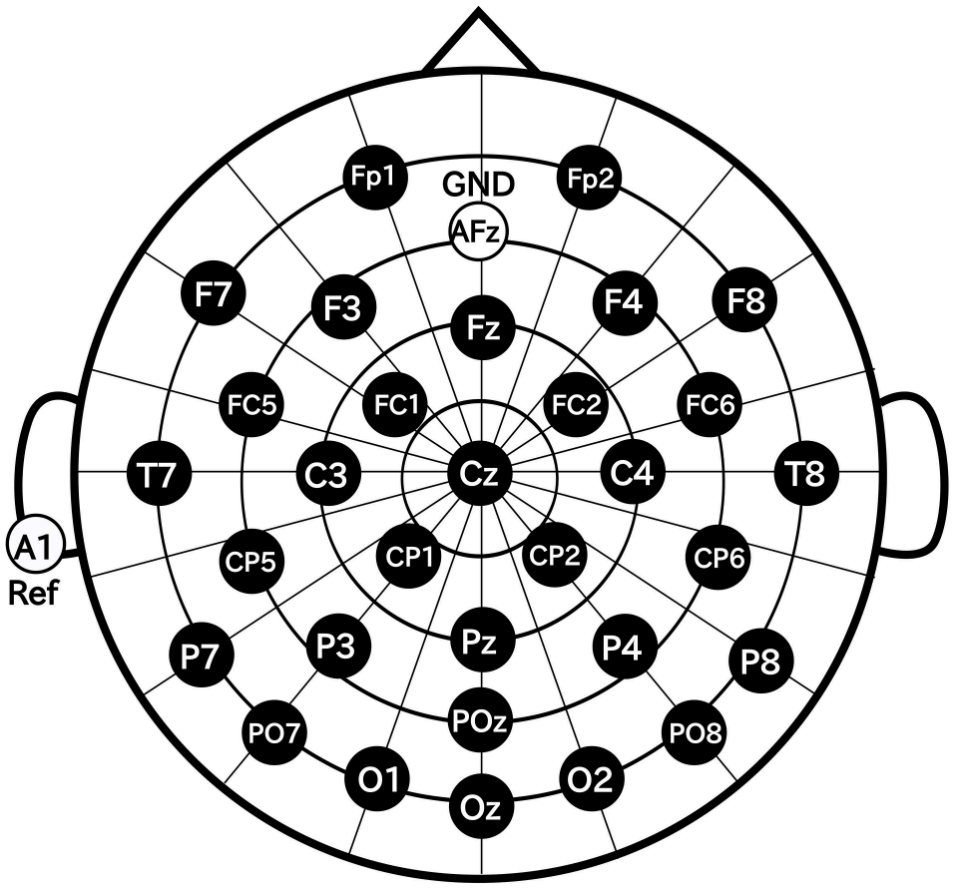
Electrode positions.

In this study, we used Ag/AgCl active electrodes which were products of Guger Technologies (g.tec) named g.LADYbird, g.LADYbirdGND (for GND), and g.GAMMAearclip (for reference, earclip type) to record EEG. These were driven by the power supply unit named g.GAMMAbox (g.tec). As shown in Figure 3, 32 electrodes were placed over the scalp in accordance with the international 10–10 system. The electrodes for GND and the reference were placed at AFz and A1, respectively. The EEG signals were amplified by MEG-6116 (Nihon Kohden) that applied low-pass and high-pass analog filters for each channel. The cut-off frequencies of the low-pass and the high-pass filters were set to 100 and 0.08 Hz, respectively. The EEG signals were sampled by A/D converter (AIO-163202F-PE, Contec) with a sampling rate of 1,024 Hz. The signals were recorded with Data Acquisition Toolbox of MATLAB (MathWorks).

### 2.4. Data analysis

#### 2.4.1. Preprocessing

We analyzed the relationship between the envelope of the sound stimuli and EEG. Thirty-four-second epochs of the EEG recordings (excluding the first 2 s after the onset of the sound stimuli and the last second before the end of them to remove filtering edge effect) were used for further analysis consisting of preprocessing and calculating a cross-correlation function.

First, a zero-phase Butterworth digital bandpass filter between 1 and 40 Hz were applied to the recorded EEG. Second, the filtered EEG were downsampled to 256 Hz. Finally, the z-score was calculated.

For the sound stimuli, a zero-phase Butterworth digital high-pass filter (1 Hz) was applied to the recorded sound stimuli. Thereafter, the envelope of the filtered sound stimuli was calculated using Hilbert transform. After that, the zero-phase Butterworth digital band-pass filter between 1 and 40 Hz were applied to the envelope. Then, the filtered envelopes were downsampled to 256 Hz. Finally, the z-score was calculated. To avoid including the brain responses evoked by the sound onset, the first second of the EEG signals and the music envelopes were discarded in the following analyses.

#### 2.4.2. Cross-correlation function

Cross-correlation function can be used to evaluate spectro-temporal characteristics of the entrainment between the stimulus and the cortical response as suggested in Lalor et al. (2009), VanRullen and Macdonald (2012). Thus, in this paper, the cross-correlations between the envelope of the sound stimuli and the EEG signals were computed as follows:

(1) $$\begin{array}{l} \text{cross}-\text{correlation}\left(\text{ch},\tau \right)=\underset{t}{\sum }\text{env}\left(t\right)\text{eeg}\left(\text{ch},t+\tau \right), \end{array}$$

where env(*t*) and eeg(*t*) denote the filtered standardized (z-scored) envelope of a sound stimulus and the corresponding filtered standardized (z-scored) EEG response at time *t* and channel *ch*, respectively. Besides, τ denotes the time lag between the envelope and EEG signal. The time lags were applied between −0.6 and 0.6 s to include the cross-correlation for a little over a second, since the band-passed signal has the minimum frequency of 1 Hz. The negative parts of the lags were used for confirming the two pronounced peaks which were commonly higher than the baseline.

#### 2.4.3. Evaluation

We conducted three evaluation tests as follows. First, in order to examine whether the cross-correlation values differs from 0 (reflecting significant phase entrainment to music stimuli), we accordingly compared our cross-correlation results with surrogate distributions by performing a statistical test in frequency domain as suggested in Zoefel and VanRullen (2016).

Second, in order to examine cross-correlation changes across the categories (familiar, unfamiliar, and scrambled) and sessions (first and second), a two-way repeated-measure analyses of variance (ANOVA) was performed. Category and session were defined as the independent variables and the two pronounced peaks (i.e., the first and the second peaks) of the standard deviation values of the cross-correlation values across the electrodes were introduced as the dependent variables (as suggested in Zoefel and VanRullen, 2016). To detect the peaks, we applied a peak detection algorithm provided by the Python Scipy library (see Supplementary Materials). As the assumption of sphericity was violated, we corrected the degrees of freedom using a Greenhouse-Geisser correction. Paired *t*-tests with Bonferroni correction for multiple comparisons were carried out as *post-hoc* analyses. The effect size was calculated as generalized eta squared ($\eta_{G}^{2}$) (Olejnik and Algina, 2003; Bakeman, 2005).

Third, in order to examine hemispheric lateralization at the two peaks across sessions by each category, a two-way repeated-measure ANOVA was performed. Electrode and session were defined as the independent variables and the two peaks of the cross-correlation values for each electrode were introduced as the dependent variables. As the assumption of sphericity was violated, we corrected the degrees of freedom using a Greenhouse-Geisser correction.

## 3. Results

We calculated the cross-correlation function between the envelope of sound stimuli and EEG for the different music categories. Thereafter, statistical tests were conducted including repeated measure ANOVA tests followed by *post-hoc* tests to analyze effects of the parameter on the cross-correlation function.

### 3.1. Experimental results

**Figure 4:**
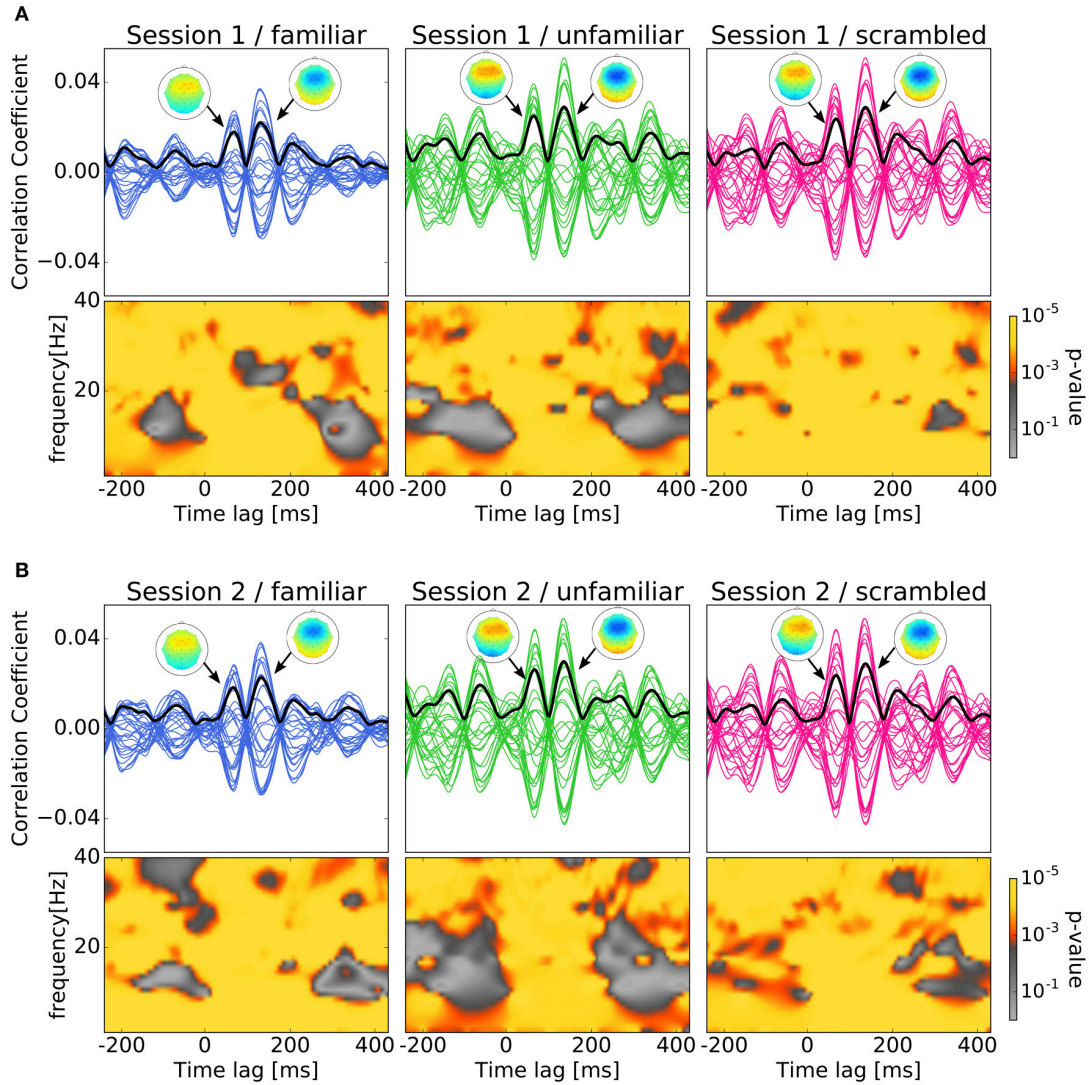
Results of cross-correlation and significance values in the time-frequency plane. In both **(A,B)**, top panels show Cross-correlation values between the envelope of sound stimuli and EEG averaged across trials and subjects for the session and category. Each line indicates the cross-correlation curve for one channel. The black solid line presents the standard deviation of the cross-correlation values across channels. Each sub-figure shows two pronounced peaks at the time lags around 70 and 140 ms. The topographies show the distribution of the cross-correlation values at the two peaks. Bottom panels show the *p*-values obtained when comparing the cross-correlation results with surrogate distributions in the time-frequency plane which show significant at all time-frequency points. **(A)** Cross-correlation values between the envelope of sound stimuli and EEG averaged across trials and subjects for the first session. **(B)** Cross-correlation values between the envelope of sound stimuli and EEG averaged across trials and subjects for the second session.

We labeled the trials which were presented the original version of the stimuli as familiar or unfamiliar category according to the participant's answers. The answers were shown in Table 1, and the number of trials used in the analysis by each category is shown in Table 2. The top panels of Figures 4A,B show the cross-correlation values between the envelope of sound stimuli and EEG for all channels averaged across the trials and the subjects in the first, and second session respectively. The black solid line presents the standard deviation of the cross-correlation values across channels for each session and each category. All categories showed two pronounced peaks at the time lags around 70 and 140 ms. The topographies show the distribution of the cross-correlation values at the two peaks for each session and category. As can be seen in Figure 4, the topographical images did not reveal any hemispheric lateralization at the two peaks. Moreover, it looks there was no difference between the sessions.

**Table 2:** Number of trials for familiar and unfamiliar category used in the analysis.

**Participant**	**Category**	
	**Familiar**	**Unfamiliar**
s1 m	10	10
s2 m	10	10
s3 m	9	10
s4 m	9	8
s5 m	10	8
s6 m	8	12
s7 m	11	7
s8 m	10	8
Mean	9.6 ± 0.92	9.1 ± 1.6

*Category was labeled according to the answer of the participant. If participants' answer are not consistent across the sessions, they were excluded from the recordings. Note that scrambled category was labeled automatically*.

In Figures 4A,B, the bottom panels show the *p*-values obtained when comparing the cross-correlation results with surrogate distributions in the time-frequency plane. As can be seen the bottom panels show significant differences at all time-frequency points. This may ensures the existence of neural entrainment to music. Indeed, it can be observed from these time-frequency spectrograms that the *p*-values at around the peak times are generally smaller than that at the other time instances.

### 3.2. Statistical verifications

First, we examine cross-correlation changes across the categories and sessions. For each peak, we performed a two-way repeated-measure ANOVA test (i.e., 2 sessions × 3 categories) on standard deviation of the cross-correlation values obtained from each subject. Summary of the results are shown in Table 3. The repeated-measure ANOVA test for the first peak (around 70 ms) yielded a significant main effect of the category, *F*_(2, 14)_ = 14.9081, *p* = 0.0009, $\eta_{G}^{2}=0.1916$, whereas there was no significant main effect of the session, *F*_(1, 7)_ = 0.1555, *p* = 0.7051, and no significant interaction of the session and the category, *F*_(2, 14)_ = 0.0114, *p* = 0.9721. Similarly, the repeated-measure ANOVA test on the second peak (around 140 ms) revealed a significant main effect of the category, *F*_(2, 14)_ = 24.7592, *p* = 0.0001, $\eta_{G}^{2}=0.1583$, whereas there was no significant main effect of the session, *F*_(1, 7)_ = 0.1642, *p* = 0.6974, and no significant interaction of the session and the category, *F*_(2, 14)_ = 1.4953, *p* = 0.2610. In summary, the results revealed that the cortical responses were significantly different between categories, while there was no difference between the cortical responses in the first and the second session.

**Table 3:** Summary of the ANOVA tests.

**Peak**	**Effect of session**	**Effect of category**	**Effect of interaction**
First peak	*p* = 0.7051	*p* = 0.0009^**^	*p* = 0.9721
Second peak	*p* = 0.6974	*p* = 0.0001^**^	*p* = 0.2610

*There were significant main effects of category in all sessions at the two peaks, and there was no significant main effect of session and interaction*.

** *p < 0.001*.

**Figure 5:**
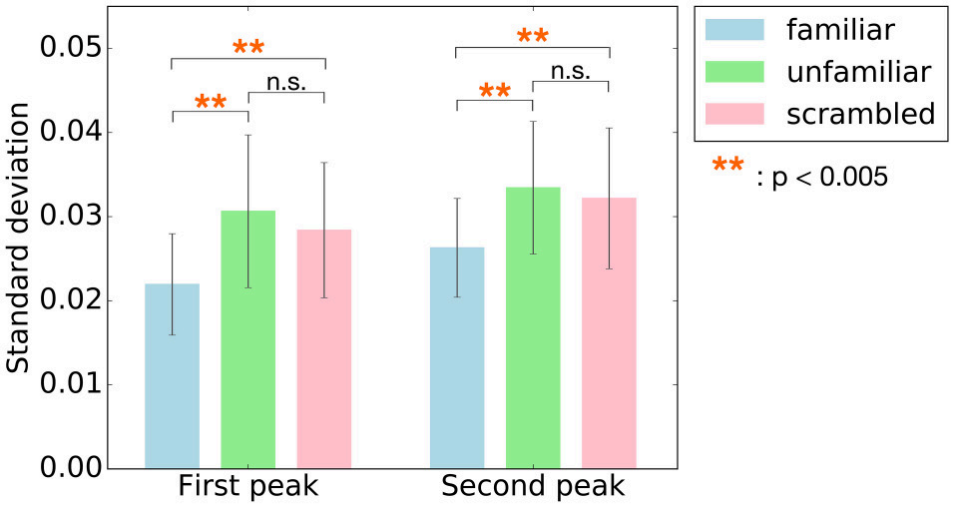
*Post-hoc* tests were performed on the main effect of the category for the two peaks, first peak (around 70 ms) and second peak (around 140 ms). The bars indicate standard deviation values of cross-correlation function averaged across subjects at the two peaks. Error bars represent standard deviation of the mean. The responses to unfamiliar and scrambled music at both two peaks were significantly stronger than to familiar music. ^**^p < 0.005.

Since the main effect of category has been observed, *post-hoc* tests were performed to better understand the changes on cross-correlation across the different categories. Summary of the results are shown in Table 3. As shown in Figure 5, paired *t*-tests showed that the responses to unfamiliar music at the first peak were significantly stronger than to the responses to familiar music, *t*_(7)_ = 4.4455, *p* = 0.0048. Moreover, the responses to scrambled music were significantly stronger than the responses to familiar music, *t*_(7)_ = 4.9826, *p* = 0.0048. Likewise, the responses to unfamiliar music at the second peak were significantly stronger than the responses to familiar music, *t*_(7)_ = 5.7120, *p* = 0.0022. Besides, the responses to scrambled music were also significantly stronger than the responses to familiar music, *t*_(7)_ = 5.0489, *p* = 0.0022. In other words, these results show that the cortical responses to unfamiliar and scrambled (i.e., non-sensical) music were stronger than the cortical responses to familiar music.

Second, in order to assess the hemispheric lateralization at the two peaks across sessions by each category, we performed a two-way repeated-measure ANOVA test. The test showed there was no significant main effect of the electrode at the first and second peak, The results show that there are no hemispheric lateralization at the two peaks.

## 4. Discussion

Our findings showed the existence of neural entrainment to music, which was supported by the *p*-values obtained from comparing the cross-correlation results and the surrogate distributions in the time-frequency plane as shown in Figure 4. Moreover, there were significant main effects of categories on the two peaks observed at standard deviations of the cross-correlation values. *Post-hoc* tests confirmed that compared to the scrambled and unfamiliar categories, the standard deviations of the cross-correlation values in the familiar category were significantly lower. This behavior was observed at both peaks. It is worthwhile to see the relation to the analysis of responses to the deviant among a sequence of familiar and unfamiliar tones. Jacobsen et al. (2005) showed that deviant tone among a sequence of familiar tones enhanced the MMN compared to that among a sequence of unfamiliar tones. This might be because that a human perceives deviant tones among a sequence of familiar tones easier. Our above-mentioned result is supportive of the finding of the previous study by Meltzer et al. (2015) which observed stronger cerebral cortex response to the periodic rhythm of scrambled (non-sensical) music compared to the familiar music. In addition to this, our results also showed that at both peaks, the standard deviation of the cross-correlation values were significantly lower in the familiar category compared to the unfamiliar category. Thus, it suggests that cortical responses to non-sensical or unfamiliar music are stronger than to the cortical responses to familiar music.

Moreover, topographical images presented at Figure 4 did not reveal any hemispheric lateralization at the two peaks in all the categories as confirmed by statistical tests. In speech perception domain, Deng and Srinivasan (2010) reported that compared to the responses to unintelligible reversed speech the responses to intelligible speech in participants left hemisphere were weaker. In music perception domain, however, Meltzer et al. (2015) showed that there were no hemispheric differences for the responses to the beat of music. In addition to this, Lamminmäki et al. (2014) reported that there were hemispheric lateralizations when listening to speech, however no hemispheric lateralization was observed when listening to tones and music. Consequently, our study along with the previous studies suggest that hemispheric lateralization could depend on the stimuli, and music perception might not have hemispheric lateralization.

Our results showed two pronounced peaks at around 70 and 140 ms in all the categories where familiarity to music has a main effect on their amplitudes. In speech perception domain, Zoefel and VanRullen (2016) compared brain responses corresponding to low- and high-level features of speech sound. They found two pronounced peaks in cross-correlation function between EEG and original (unprocessed) speech. On the other hand, the earlier peak was much less evident when participants listened to constructed (speech/noise mixture) stimuli including only high-level acoustic features of speech. Consequently, they suggested that the earlier peak reflected low-level features whereas the later peak underlay high-level features. In our experiment, we investigated high-level factors of music perception which link to familiarity. Interestingly, in both familiar and unfamiliar music the observed two peaks were evident. Based on the studies reporting that the processing of the structure in music and speech are different (Farbood et al., 2015), our results indicate that both two peaks could be linked to high-level factors of music perception. In fact, further studies are needed to better understand how human perceives music and speech in terms of high-level and low-level factors.

In summery, this paper investigated the relationship between cortical response and familiarity of music using melodies produced by piano sounds as simple natural stimuli. The standard deviations of the cross-correlation values at the two peaks when listening to the unfamiliar and the scrambled music were significantly larger than that of listening to the familiar music. This finding suggests that the cortical response to music could be stronger to unfamiliar music than to familiar music.

Similar to other studies, there are some limitations in this study. First, all sound stimuli used in this study had the same tempo, and used only one single tone and single instrument. Second, the brain responses were recorded using EEG which is known to have low resolution. MEG would provide us clearer findings due to its higher resolution. In addition, the analysis is based on a small number of subjects. Regardless of all these limitations our results are encouraging to do further studies in future to better understand the mechanism of music perception in brain. One potential application of our technique is music therapy to enhance different brain states. It would be also possible to use it in music lessons to assess the performance. Further our tool can be implicated in neuromarketing such as music recommendation services using EEG as personalized wearable device.

## Author contributions

YK designed the experiment, collected data, contributed to analysis and interpretation of data, and wrote the initial draft of the manuscript. MA have contributed to data analysis and interpretation, and critically reviewed the manuscript. TT designed the experiment, contributed to analysis and interpretation of data, and revised the draft of the manuscript. The final version of the manuscript was approved by all authors.

### Conflict of interest statement

The authors declare that the research was conducted in the absence of any commercial or financial relationships that could be construed as a potential conflict of interest.
