# Supplementary material for: Familiarity Affects Entrainment of EEG in Music Listening
Source: Front Hum Neurosci. 2017 Jul 26;11:384. doi: 10.3389/fnhum.2017.00384 (PMC5526927; doi:10.3389/fnhum.2017.00384)
Supplement: Supplementary file 1 [file Presentation1.PDF]

# *Supplementary Material*: Familiarity affects entrainment of EEG in music listening

**Yuiko Kumagai, Mahnaz Arvaneh and Toshihisa Tanaka***
*Correspondence:
Toshihisa Tanaka
tanakat@cc.tuat.ac.jp

## 1 LIST OF AUDIO FILES

Examples of the sound stimuli listed in Table 1 of the main manuscript are provided as audio files. The list of these music pieces are as follows:

| | |
|---|---|
| Audio 1 | Twinkle Twinkle Little Star (familiar) |
| Audio 2 | Piano Sonate Op.82 (unfamiliar) |
| Audio 3 | Twinkle Twinkle Little Star (scrambled) |

## 2 LATENCY OF THE FIRST, SECOND, AND THIRD PEAKS

Table below presents the average of peak time (i.e. peak latency) and their standard deviations across the subjects for the three largest peaks in different categories and sessions.

| | Familiar | | Unfamiliar | | Scrambled | |
|---|---|---|---|---|---|---|
| Peak | Session 1 | Session 2 | Session 1 | Session 2 | Session 1 | Session 2 |
| First | $66.4 \pm 6.18$ | $64.5 \pm 6.48$ | $64.5 \pm 8.51$ | $67.4 \pm 8.46$ | $67.9 \pm 8.27$ | $68.8 \pm 8.04$ |
| Second | $131.8 \pm 10.1$ | $133.8 \pm 11.2$ | $134.8 \pm 5.17$ | $135.2 \pm 4.76$ | $137.2 \pm 6.89$ | $133.8 \pm 6.40$ |
| Third | $220.2 \pm 25.1$ | $223.6 \pm 29.3$ | $227.1 \pm 24.5$ | $240.2 \pm 27.8$ | $221.2 \pm 22.3$ | $228.5 \pm 21.9$ |

Moreover, to investigate the effect of outliers, we created boxplots of the average peak latencies across the subjects (Figure S1). The table and Figure S1 illustrate that the time that the first and second peaks are observed were more consistent across the subjects than that at the third peak. In the other words, we observed less variations in the average latency of the first and the second peaks compared to the average latency of the third peaks.

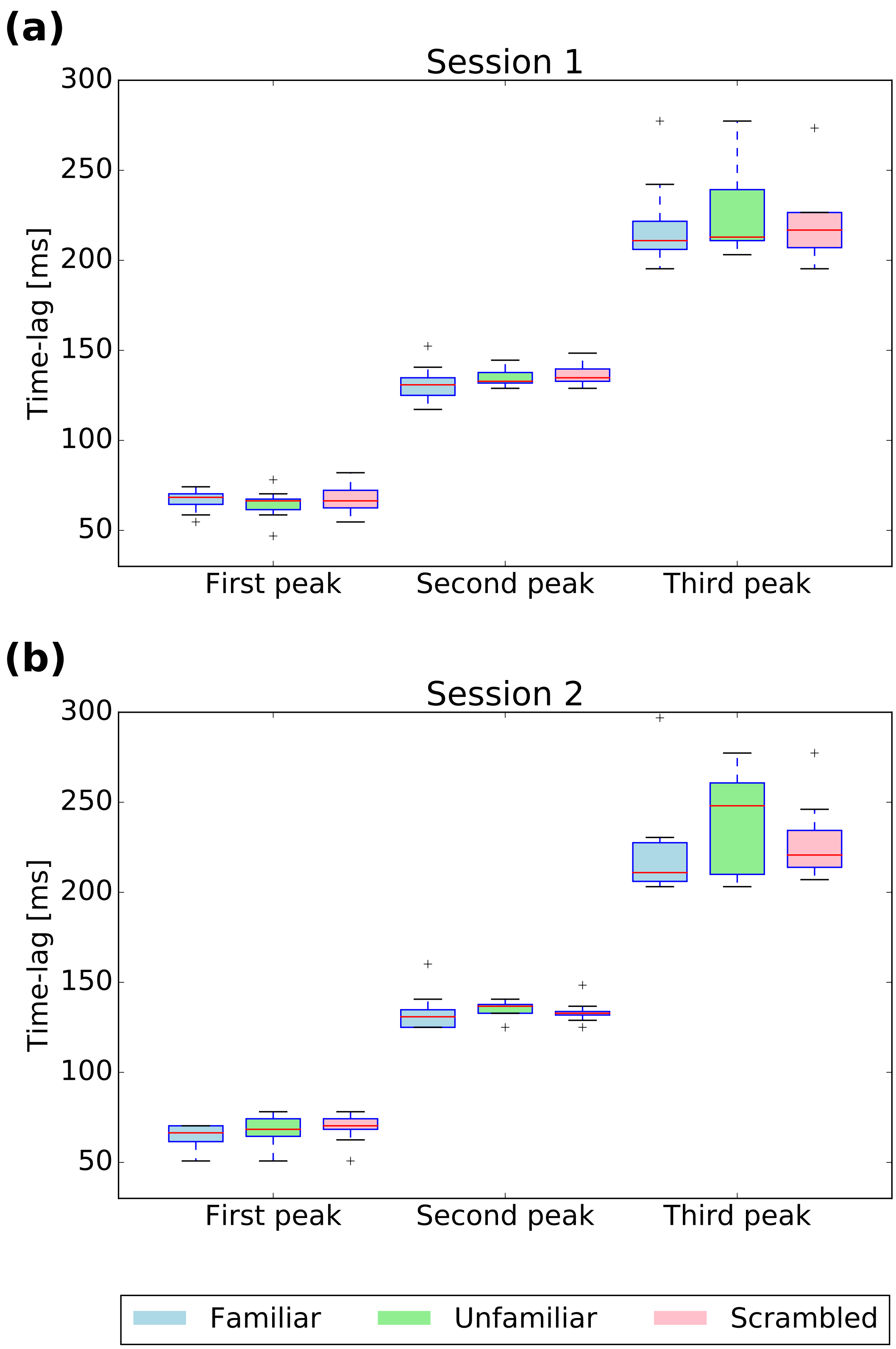


**Figure S1.** Boxplots of the average peak time of the participants for three largest peaks. The average time that the first and second peaks are observed were more consistent across the subjects compared to the average time of the third peak.
